# Combining Genetic and Phenotypic Analyses for Detecting Bread Wheat Genotypes of Drought Tolerance through Multivariate Analysis Techniques

**DOI:** 10.3390/life14020183

**Published:** 2024-01-25

**Authors:** Mohammed Sallam, Abdelhalim Ghazy, Abdullah Al-Doss, Ibrahim Al-Ashkar

**Affiliations:** Department of Plant Production, College of Food and Agriculture Sciences, King Saud University, Riyadh 11451, Saudi Arabia; msallam@ksu.edu.sa (M.S.); aghazy@ksu.edu.sa (A.G.); aaldoss@ksu.edu.sa (A.A.-D.)

**Keywords:** genetic diversity, SSR markers, mantel test, multivariate analysis

## Abstract

Successfully promoting drought tolerance in wheat genotypes will require several procedures, such as field experimentations, measuring relevant traits, using analysis tools of high precision and efficiency, and taking a complementary approach that combines analyses of phenotyping and genotyping at once. The aim of this study is to assess the genetic diversity of 60 genotypes using SSR (simple sequence repeat) markers collected from several regions of the world and select 13 of them as more genetically diverse to be re-evaluated under field conditions to study drought stress by estimating 30 agro-physio-biochemical traits. Genetic parameters and multivariate analysis were used to compare genotype traits and identify which traits are increasingly efficient at detecting wheat genotypes of drought tolerance. Hierarchical cluster (HC) analysis of SSR markers divided the genotypes into five main categories of drought tolerance: four high tolerant (HT), eight tolerant (T), nine moderate tolerant (MT), six sensitive (S), and 33 high sensitive (HS). Six traits exhibit a combination of high heritability (>60%) and genetic gain (>20%). Analyses of principal components and stepwise multiple linear regression together identified nine traits (grain yield, flag leaf area, stomatal conductance, plant height, relative turgidity, glycine betaine, polyphenol oxidase, chlorophyll content, and grain-filling duration) as a screening tool that effectively detects the variation among the 13 genotypes used. HC analysis of the nine traits divided genotypes into three main categories: T, MT, and S, representing three, five, and five genotypes, respectively, and were completely identical in linear discriminant analysis. But in the case of SSR markers, they were classified into three main categories: T, MT, and S, representing five, three, and five genotypes, respectively, which are both significantly correlated as per the Mantel test. The SSR markers were associated with nine traits, which are considered an assistance tool in the selection process for drought tolerance. So, this study is useful and has successfully detected several agro-physio-biochemical traits, associated SSR markers, and some drought-tolerant genotypes, coupled with our knowledge of the phenotypic and genotypic basis of wheat genotypes.

## 1. Introduction

Wheat (*Triticum aestivum* L.) is native to southwest Asia and is cultivated on a worldwide scale [1]. It is estimated that wheat is the second most important and widely cultivated crop in the world [2]. It is used as a staple crop by a third of the world’s population and is referred to as the “king of grains” given its importance [3]. Grains are rich in carbohydrates, making them a good source of energy [4,5]. Agricultural productivity could fall dramatically due to extreme environmental events, such as reduced water availability, which is a serious concern for the entire Arab region [1,6]. This results in more adverse effects threatening the sustainability of production from grain crops, coupled with the inferior quality. The challenge is increased because of the loss of arable land to sustainable urbanization and the steady rise in population, coupled with the declining availability of natural resources owing to climate change, which represents a serious threat to the safe production of wheat [6,7,8]. For those reasons, we aim to constantly increase productivity by 2–3% each year by targeting breeding efforts toward increasing wheat productivity through the provision of improved varieties that are high-yielding and tolerant to drought, coupled with other environmental stresses, to replace degraded varieties [1,6,9].

Drought stress affects plants differently during each stage of growth, causing many physiological and biochemical problems, leading to the overproduction of reactive oxygen species (ROS) in plants, which negatively affects the morphological and physiological characteristics of wheat crops, including plant height, leaf area, relative water content, stomatal conductance, chlorophyll content, osmotic capacity, leaf water potential, and the final product, which is the yield according to [2,10,11,12]. The production of ROS is dangerous, as it causes significant damage to cellular organelles, such as mitochondria, nucleic acids, membrane lipids, chloroplasts, and metabolic enzymes in plant cells [12]. This causes an imbalance in the physiological and biochemical processes that lead to cell death during oxidative stress induced by dehydration [13,14]. Damage to photosynthesis and desiccation-induced stomatal closure are some of the most sensitive activities to drought stress [15]. According to reports, closing the mouth reduces photosynthesis, which in turn reduces the amount of carbon dioxide available [15,16]. Therefore, when searching for genotypes that are tolerant to drought, it is very important to study the antioxidants, proline and glycine betaine, which have a significant role in removing oxygen-free radicals that cause damage to plant cells and their components. These parameters directly and/or indirectly affect different stages of plant growth, making them important for helping plant breeders choose genotypes that are drought tolerant [1,17].

Drought tolerance is a complex genetic trait regulated by a large number of genes. In addition to being genetically unstable due to genotypes being affected by their interaction with the environment that surrounds them, it is a difficult and complex process. The selection and production process of high-yielding and drought-tolerant genotypes are the basis for overcoming drought-related problems and are essential to ensure sustainable food production [2,7,18]. So, plant breeders face several key challenges in achieving this goal through close cooperation with researchers and experts in relevant areas [19]. Most recently, a molecular DNA marker was used, showing promise in facilitating and enhancing sustainable agriculture since it can provide renewable genetic inputs [1,20]. These markers have been used in many studies (genetic diversity, molecular-assisted selection (MAS), paternity analysis, mapping of quantitative trait loci, cultivar identification, phylogenetic relationship analysis, and genetic mapping), and DNA fingerprinting markers played a major in the early detection of polymorphisms [17,21,22,23]. The selection process is more accurate and fully transparent when genetic markers (not influenced by environmental factors) work alongside phenotypic traits (influenced by environmental factors) and can be genotype-assessed accurately and more objectively by markers required to produce a specific pattern of bands for each individual [7,17,24].

SSR markers are one of the main molecular markers characterized by multi-allele, co-dominant inheritance between generations, information-rich, relatively highly abundant, distributed on a massive scale across the genome, and potential for replication [1,7,17,25]. SSRs are very beneficial for various studies in genetics and breeding, assisted selection for crop improvement, and genetic diversity estimation, coupled with population structure and gene mapping analyses [17,21,26,27]. Therefore, SSR markers are a suitable choice for genetic diversity studies of wheat genotypes. Some recent studies have highlighted using SSR markers, which have succeeded in wheat genetic diversity analysis and shown that genotypes have diverged to a large extent—an essential consideration in breeding programs for drought tolerance [21,25,28,29,30,31]. The discovery of QTLs (quantitative trait loci) has made a revolution in the selection process for genes conferring quantitative traits (such as drought tolerance) in MAS, which are mostly located in the A and B genomes on chromosomes 2B, 3A, 4A, 4B, 7A, and 7B [7,8,28,32]. There is, therefore, an urgent need to integrate molecular tools with precise high-throughput phenotyping to confirm their interdependence [17,33].

Statistical analyses are required to obtain accurate selection criteria and efficient screening methods in breeding programs. Screening tests of large amounts of data require appropriate statistical analysis to formulate conclusions and make recommendations concerning genotypes that are tolerant and/or sensitive [14,34]. Naturally, the performance of agro-physio-biochemical and molecular responses will differ from one genotype to another, so it is superior in some traits and lesser in other traits [1,17,24]. The aim of this study is to assess the genetic diversity of 60 genotypes using SSRs collected from several regions of the world and select more genetically diverse genotypes for studying drought stress. The effective use of multivariate analyses and divergent traits serves as confident screening criteria for evaluating genotypes under drought stress. Furthermore, this study intends to obtain genotypes that are highly drought-tolerant and high-yielding.

## 2. Materials and Methods

### 2.1. Genomic DNA Extraction and SSR Markers

Sixty genotypes were used for screening the genetic diversity of drought-tolerance genotypes (35 genotypes obtained from CIMMYT (International Maize and Wheat Improvement Center), twenty-two genotypes obtained from Professor Abdullah Al-Doss, two genotypes obtained from the Agricultural Research Center, Egypt, and one genotype (DHL2) obtained from Professor Ibrahim Al-Ashkar) (Table S1). All genotypes were germinated in growth chambers, with the seeds placed on Petri dishes. Once the plants reached the fourth leaf stage, the leaves were collected for DNA extraction. Genomic DNA extraction for all genotypes followed the CTAB procedure, as described by Saghai-Maroof et al. [35]. To determine the quantity and quality of the extracted DNA, UV–vis spectrophotometry was employed, measuring the absorbance at 260 nm. Additionally, electrophoresis using a 0.8% agarose gel was conducted to assess the DNA samples further. Eighty SSR markers associated with drought stress in wheat were selected, according to many scientists [36,37,38,39,40,41,42].

PCR reactions were conducted following the protocol [43] utilizing a 96 thermocycler. Each PCR reaction was performed in a 20 μL volume, using Promega Green master mix, which consists of dNTPs, Taq polymerase, and a 10× PCR buffer. The reaction mixture also contained 1 mM MgCl_2_, 15 pmole of each primer, and 100 ng of genomic DNA as the template. After PCR amplification, the resulting products were subjected to electrophoresis on 3% agarose gels. To visualize the DNA fragments, the gels were stained with ethidium bromide (EtBr) and placed in a Gel doc system. A 100 bp ladder was used as a size standard for comparison. The DNA fragments separated in the agarose gel were visually examined and scored. Each fragment, also known as an allele, was scored as either 1 (indicating its presence) or 0 (indicating its absence) for each marker.

### 2.2. Plant Materials and Experimental Design

Thirteen genotypes (DHL2, 16HTWYT-22, KSU115, 16HTWYT-38, KSU105, Yecora Rojo, Klassic, ksu106, 16HTWYT-30, 16HTWYT-20, 16HTWYT-9, 16HTWYT-12, and Line-47) were selected between tolerant and sensitive depending on the results of SSR markers for further phenotypic and genetic analyses of studied traits. In the 2021/22 growing season, these thirteen genotypes were planted in rows measuring 2.0 m in length, with a spacing of 0.20 m between rows in three repetitions using a randomized complete block design for separate irrigation (I) treatments (control (C) and drought (D)). Fertilization rates were 3.1 g m^−2^ P_2_O_5_ (with the tillage operation) and 18 g m^−2^ N (during watering in batches). The drought treatment was applied two weeks after sowing (irrigation when soil moisture was at thirty-three percent of the field capacity), and the control treatment was applied (irrigation when soil moisture was at eighty percent of the field capacity).

### 2.3. Measurements of Traits

#### 2.3.1. Agro-Physio-Biochemical Traits

Twenty-six agro-physio-biochemical traits were studied (six physiological traits, twelve Agronomic traits, and nine biochemical traits). The physiologic traits (relative water content (RWC), relative turgidity (RT), net photosynthetic rate (Pn), stomatal conductance (Gs), intercellular carbon dioxide (Ci), and transpiration rate (E)) and the agronomic traits (days to heading (DH, days), days to maturity (DM, days), duration of grain filling (GFD, day), grain filling rate (GFR g/day), plant height (PH, cm), flag leaf area (FLA) and spike length (SL, cm), spikes per square meter (NS, m^2^), spikelets per spike (NSS, per spike), grain yield (GY, ton ha^−1^), and thousand-kernel weight (TKW, g)) were measured as explained in detail by Al-Ashkar et al. [2]. The biochemical traits (chlorophyll content (Chl), superoxide dismutase (SOD), polyphenol oxidase (PPO), catalase (CAT), peroxidase (POD), 2,2-diphenyl-1-picrylhydrazyl radical (DPPH), total phenolic content (TPC), Glycine betaine (GB), and proline content (Pro)) measured as shown below:

- The estimation of Chl in leaves was performed using a colorimetric method by measuring absorbances at 663 nm and 646 nm with 80% acetone as the solvent. Approximately 0.5 g of leaf tissue was crushed in liquid nitrogen, and about 100 mg of the crushed tissue was taken. Then, 2 mL of acetone was added to the sample, which was then left in a dark place in the refrigerator for 48 h. Afterward, the sample was centrifuged, and the extract obtained was used for spectrophotometer readings to estimate Chl. The calculations for estimating Chl were based on the equations provided by Lichtenthaler and Wellburn [44].
- To measure the activity of antioxidant enzymes, including SOD, CAT, POD, and PPO, fresh leaf samples weighing 0.5 g were utilized. The extraction of these enzymes involved crushing the leaves in liquid nitrogen and suspending them in a buffer containing 50 mM potassium phosphate buffer (pH 7.8) and 1% (*w*/*v*) polyvinyl polypyrrolidone. Afterward, the samples were subjected to centrifugation at 14,000× *g* for 10 min at 4 °C. The resulting supernatant, as described in references [45,46,47], was used as an enzyme extract for the subsequent tests assessing the activity of CAT, POD, PPO, and SOD.
- The determination of DPPH radical scavenging ability involved assessing the decrease in absorbance at 517 nm [48]. The analyses were conducted using a UV–vis spectrophotometer in 3 mL cuvettes. To facilitate the analysis, a freshly prepared stock solution of DPPH (3.94 mg/100 mL methanol) radicals in methanol was utilized. Subsequently, 3 mL of the DPPH working solution was mixed with 0.5 mL of the extract and left in darkness for 30 min. The presence of an antioxidant agent in the reaction medium led to the disappearance of the purple color associated with DPPH radicals. In parallel, a reference sample consisting of 0.5 mL of the solvent was prepared. The maximal absorption of the newly prepared DPPH radical solution was observed at 517 nm. All analyses were performed in 3 replicates, and the absorbance was recorded at 517 nm. The blank sample referred to the reaction mixture that lacked any test compounds [49,50]. DPPH scavenging effect (%) = [A_0_ − A_1_)/A_0_] × 100.
- The quantification of TPC was conducted using the Folin–Ciocalteau method, as previously described by Sarker and Oba [51]. In this procedure, extracts (100 μL) or a series of standards (12.5, 25, 50, 100, 150, and 200 μg mL^−1^ gallic acid) were added. Following reagent mixing and the ensuing reaction, 300 μL of the solution was transferred to a 96-well plate, and the absorbance was measured at 740 nm. The obtained results were expressed as the equivalent amount of gallic acid standard (mg GAE/g FW).
- To quantify the contents of GB, leaf samples were ground using liquid nitrogen to ensure proper homogenization, as described by Grieve and Grattan [52]. Subsequently, 1 mg of the sample was transferred to a glass tube, and 1.5 mL of 2 N H_2_SO_4_ was added to it. The mixture was then placed in a water bath at 60 °C for ten minutes to extract Glycine betaine. After centrifugation at 3500× rpm for 10 min, the supernatants were collected for further analysis. To analyze the GB concentration, 125 μL of the supernatant sample was combined with 50 μL of cold Potassium tri-iodide KI-I2, which was prepared by dissolving 15.7 g of iodine and 20 g of potassium iodide in 100 mL of distilled water. This mixture was left at 0–4 °C for 16 h and then centrifuged at 10,000× rpm for 15 min. The upper liquid was discarded, leaving behind small crystals in the chamber of the tube. These crystals were dissolved by adding 1.4 mL of 1,2-dichloroethane and incubating the solution for 2–2.5 h. The samples were then examined using a spectrophotometer at 365 nm (U-2000, Hitachi Instruments, Tokyo, Japan). To determine the GB concentration, a standard curve was prepared using stock solutions of betaine with concentrations of 1, 2, 4, 6, and 8 μL. These stock solutions were used to calculate the GB concentration in the samples.
- For proline content, the estimation of proline was performed using the protocol described by Boctor [53] with certain modifications. Initially, the sample was ground using liquid nitrogen. Then, 100 mg of the sample was taken and mixed with 500 μL of 3% Sulpho salicylic acid. The mixture was vortexed and placed on ice for five minutes, followed by centrifugation at the highest speed for five minutes at room temperature. Next, 200 μL of the supernatant was combined with 200 μL of 3% Sulphosalicylic acid, 400 μL of glacial acetic acid, and 400 μL of ninhydrin acid. The reaction components were vortexed thoroughly and placed in a water bath at 100 °C for one hour. To halt the reaction, the tubes were then placed on ice. In the final step, 1 mL of toluene was added to the reaction mixture. The solution was vigorously shaken by hand and left undisturbed for five minutes to allow the components to separate into two layers. The top layer was extracted, and the absorbance was measured using a spectrometer at 520 nm, with toluene serving as the blank.

#### 2.3.2. Quality Traits

Four traits (protein content (pc), wet gluten content (WGC), dry gluten content (DGC), and gluten index (GI)) were estimated as explained in detail by the Committee [54].

### 2.4. Statistical Analysis

- Genotyping Analysis: SSR bands were scored (present (1) or absent (0)) to create a binary matrix. The genetic dissimilarity (matrix of pairwise) between genotypes was calculated using the coefficient of Jaccard dissimilarity. Agglomerative HC analysis was implemented using the unweighted pair group average method (UPGAM).
- Phenotypic analysis: ANOVA (split-plot design) and genetic parameters for 30 traits were implemented using SAS v9.2 software (SAS Institute, Inc., Cary, NC, USA). The variance (mean squares) of data for 30 traits was used to compute variance components that are used to compute genetic parameters (genetic variance (σ^2^G), residual variance (σ^2^e), phenotypic variance (σ^2^Ph), heritability (h^2^ %), genotypic coefficient of variability (G.C.V. %), phenotypic coefficient of variability (Ph.C.V. %), genetic advance (GA), and genetic gain (GG)), as described by Al-Ashkar et al. [14]. Principal component analysis (PCA) was carried out based on data provided by the correlation matrix to find out the variables contributing the most to the variance and the components loading the most on the variables. PCA is useful for trait reduction, dealing with the problem of multicollinearity, and identifying important traits that are located in the first two components, and its outcomes were used to detect the drought tolerance index, which was used in SMLR (Stepwise multiple linear regression), PC (path coefficient), HC (hierarchical cluster), and LD (liner discriminant) analyses. PCA eliminated five traits that exhibited high multicollinearity. Twenty-five out of thirty traits (index) were used in SMLR to determine the key traits that contribute to enhancing and developing the variable of interest (GY), after which PC analysis was used to divide variation into direct and indirect effects. The effective indices (nine out of twenty-five traits) were used in the HC analysis to evaluate the genetic dissimilarity matrix between thirteen genotypes, characterized into three tolerance groups using Euclidean distance and Ward’s method of agglomeration. LD Analysis was employed to validate the genotype tolerance categories (the nine indices used as quantitative variables) with the three categories (as qualitative variables). Statistical analyses (PCA, SMLR, PC, HC, and DFA) were implemented through XLSTAT statistical package software (vers. 2019.1, Excel Add-ins soft SARL, New York, NY, USA).

## 3. Results

### 3.1. Screening Genetic Diversity of Drought Tolerance Genotypes

**Figure 1:**
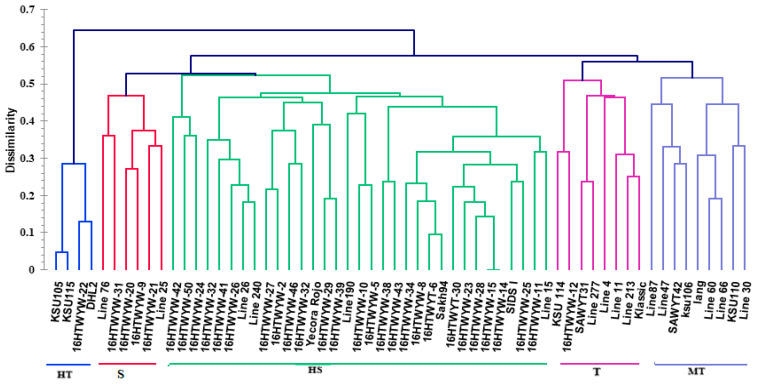
Dendrogram showing clustering of 60 wheat genotypes based on SSR markers.

The microsatellite (SSR) data were used to determine genetic dissimilarities (based on levels of tolerant drought) for 60 genotypes according to the Jaccard coefficient. The hierarchical cluster (HC) analysis divided genotypes into five main categories: The first category (HT, high tolerant) covered four genotypes (16HTWYW-22, KSU105, KSU115, and DHL2) (Figure 1). The second category (T, tolerant) covered eight genotypes (KSU 114, SAWYT31, Line 277, Line 4, 16HTWYW-12, Line 213, Klassic, and Line 11). The third category (MT, moderate tolerant) covered nine genotypes (Line 87, KSU110, Line 30, lang, Line 47, Line 60, Line 66, SAWYT42, and ksu106). The fourth category (S, sensitive) covered six genotypes (Line 76, 16HTWYW-21, 16HTWYW-31, 16HTWYW-20, 16HTWYW-9, and Line 25), while the fifth category (HS, high sensitive) covered 33 remaining genotypes. Thirteen genotypes were selected depending on the results of SSR markers for further phenotypic and genetic analyses of studied traits.

### 3.2. Phenotypic Analysis of Genotypes and Traits

#### 3.2.1. ANOVA, Genetic Parameters, and Genotype Performance

**Figure 2:**
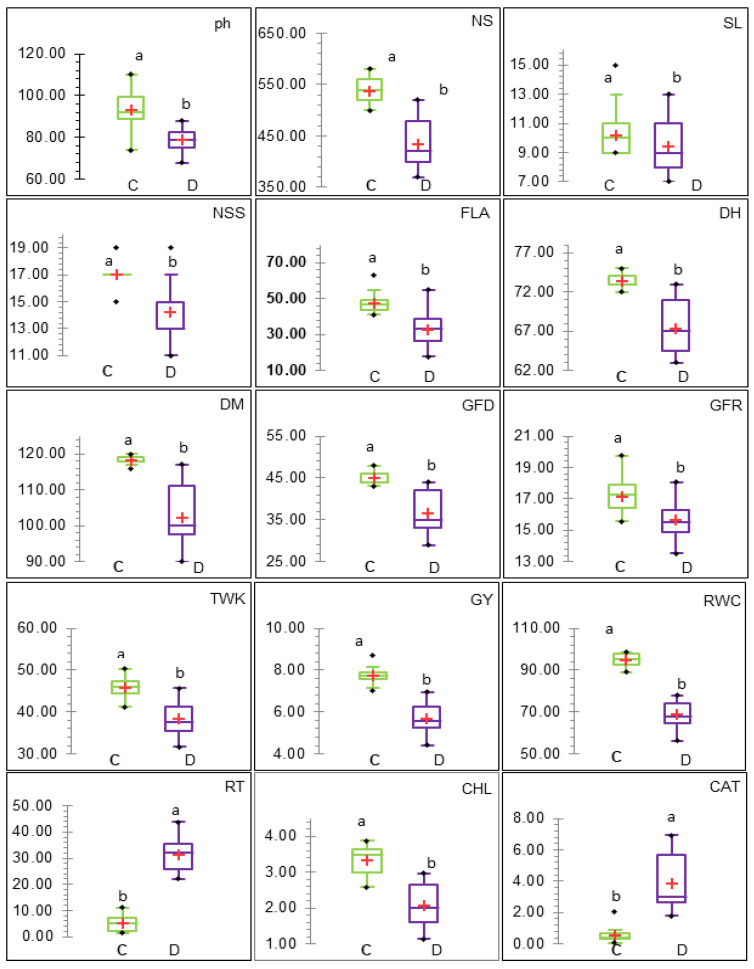

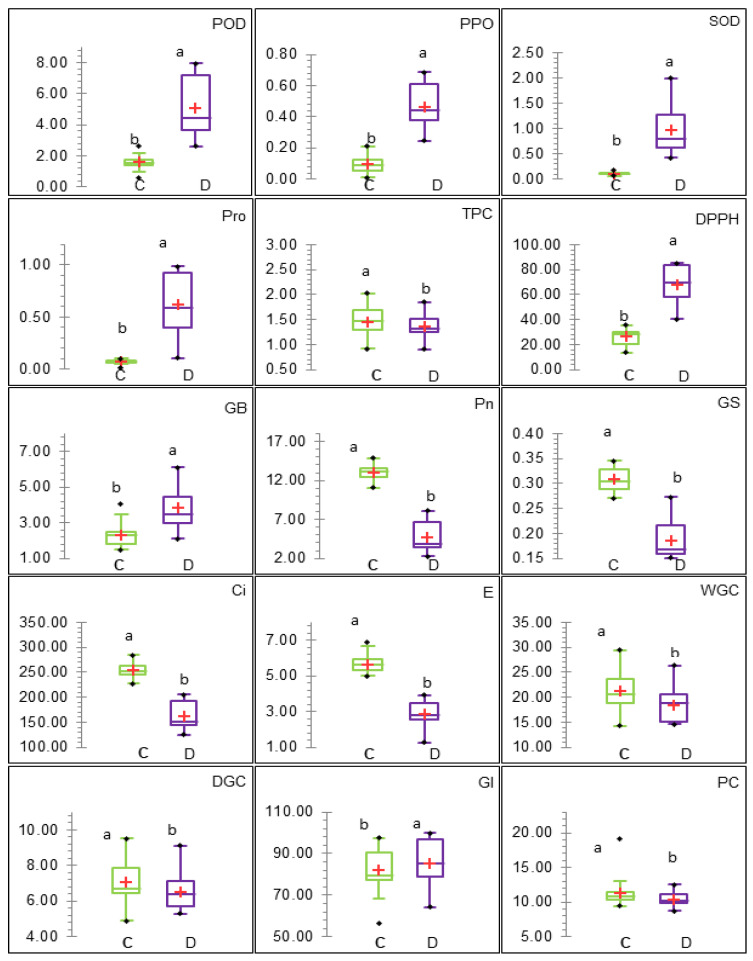
Boxplots illustrating the descriptive statistics of 30 measured traits under control (C) and drought (D) conditions for 13 wheat genotypes. Letters a and b indicate significant differences based on the Duncan test at the 0.01% level. Abbreviations for traits are as described in materials and methods. The numbers on the Y-coordinate axis indicate measurement trait units.

ANOVA detected highly significant (*p* < 0.01) variations between the two (optimal and drought-stressed) treatments (I) in 25 measured traits, significant (*p* < 0.05) variations in three traits, and insignificant in two traits. The genotypes (G) and interaction (I × G) showed highly significant (*p* < 0.01) variations for 30 measured traits (Table 1). The (h^2^) showed high values (>60%) for 15 traits [60.57% ≤ h^2^ ≤ 96.459%] and moderate for 14 traits [57.42% ≤ h^2^ ≤ 38.72%]. The (GG) showed high values (>20%) for 11 traits [61.58% ≤ GG ≤ 20.03%] and moderate for eight traits [16.06% ≤ h^2^ ≤ 10.62%]. The PCV and GCV values were convergent for some traits and divergent for some other traits. The σ^2^G was smaller than the σ^2^Ph for all traits. The mean values of 21 traits studied in the optimal treatment (C) were greater than the drought-stressed treatment (D), except for nine physio-biochemical traits (RT, CAT, POD, PPO, SOD, Pro, DPPH, GB, and GI), as shown in Figure 2. The maximum values were such that the mean values, which were recorded for the same traits [13 traits (C) were greater than (D) and 9 traits (D) were greater than (C)].

#### 3.2.2. Multidimensional Analyses in the Classification of Drought-Tolerant Genotypes

##### Principal Component Analysis (PCA)

**Figure 3:**
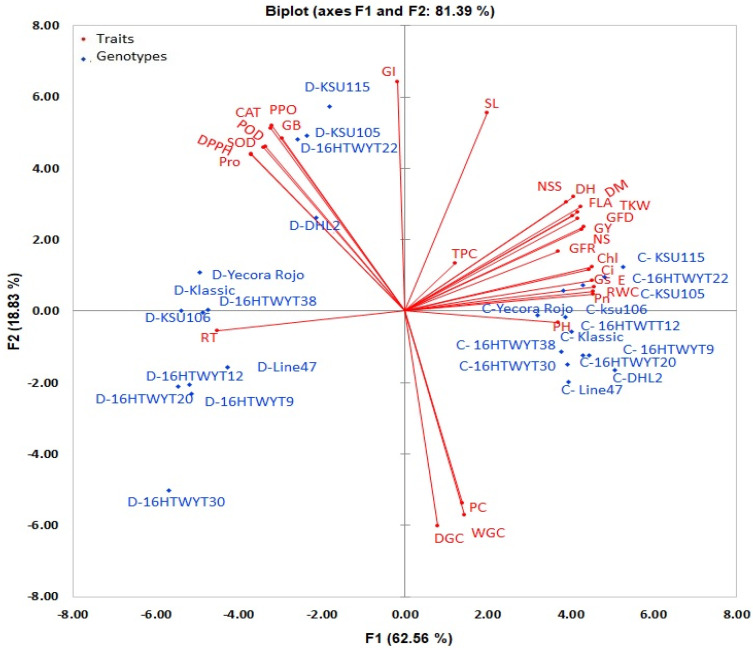
Biplot for the first two principal components in the principal component analysis of 13 wheat genotypes. Abbreviations as described in Section 2. The genotypes started with C and D means the control and drought conditions, respectively.

The first four PCAs showed eigenvalue >1, which explained 89.79 of the total variances for the 30 traits studied. PCA1 and PCA2 contributed to explaining 62.56% and 18.83% of the total variance, respectively. PCA1 (values of ≥0.381) was related to 24 traits (RWC, RT, Chl, CAT, POD, PPO, SOD, Pro, DPPH, GB, Pn, Gs, Ci, E, FLA, DH, DM, GFD, GFR, PH, NS, NSS, TKW, and GY). PCA2 was related to five traits (SL, WGC, DGC, GI, and PC). PCA3 was not related to any trait, and PCA4 was related to the TPC trait (Table 2). Figure 3 shows the correlations between traits on PCA1 and PCA2, which explained 25 out of 30 traits (five traits were not included due to collinearity). PCA1 had a positive correlation with sixteen and a negative correlation with nine traits, while PCA2 had a positive correlation with twenty and a negative correlation with five traits. All genotypes under drought exhibited negative correlations with PCA1, and five of them exhibited negative correlations with PCA2.

##### SMLR and PC Analyses for the Performance of Yield Trait

Since the major purpose is the yield, we used both SMLR and path coefficient analysis to identify genetically influenced traits. The results from SMLR indicated that the contribution rates of FLA, Gs, PH, and RT were 0.870, 0.049, 0.056, and 0.014, respectively, for a total of 0.988 (the residual value was 0.109) (Table 3). Given the substantial correlation of FLA with yield, we searched for the traits associated with it, which were GB, PPO, Chl, and GFD, with contribution values of 0.827, 0.059, 0.048, and 0.040, respectively, for a total of 0.975 (the residual value was 0.158). In PC analysis, the four traits of GY were divided into direct and indirect effects. FLA alone contributed 0.464 (as a direct effect) out of 0.988, and total direct and indirect impacts for the four traits were 0.681 and 0.307, respectively. If the FLA trait was its dependent variable, the GB trait alone contributed 0.437 (as a direct effect) out of 0.975, and total direct and indirect impacts for the four traits were 0.634 and 0.341, respectively.

##### Hierarchical Clustering and Linear Discriminant Analyses

**Figure 4:**
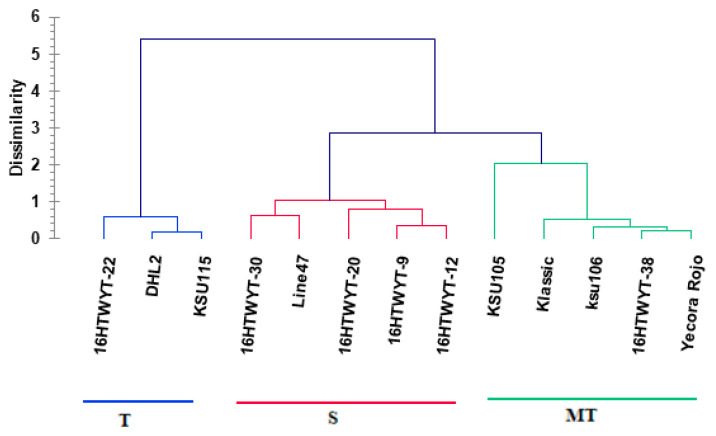
Dendrogram showing clustering of 13 wheat genotypes based on Euclidean distance.

Based on SMLR and PC analyses, we used the nine indices (FLA, Gs, PH, RT, GB, PPO, Chl, GY, and GFD) for HC analysis to assess drought tolerance in the 13 wheat genotypes used. HC analysis divided genotypes into three main categories based on nine tolerance indices. The first category (T, tolerant) covered three genotypes (DHL2, 16HTWYT-22, and KSU115). The second category (MT, moderate tolerant) covered five genotypes (16HTWYT-38, KSU105, Yecora Rojo, Klassic, and ksu106). The third category (S, sensitive) covered five genotypes (16HTWYT-30, 16HTWYT-20, 16HTWYT-9, 16HTWYT-12, and Line47), as shown in Figure 4. 

LD analysis was used to strengthen the reliability of the categories. The category of the three groups (T, MT, and S) (prior and posterior) was verified. LD analysis showed that the prior and posterior categories were completely identical in the thirteen genotypes used (% correct = 100%). Membership probability values (>0.5) are indicative of the extent of compatibility between prior and posterior categories, with membership probability values = 1 for all thirteen genotypes used (Table 4). The compatibility proportion differed cross-validation of prior and posterior categories, and it was compatible in nine genotypes (% correct = 69.23%) and incompatible in four genotypes (16HTWYT-30, 16HTWYT-20, 16HTWYT-12, and 16HTWYT-22). The Mahalanobis distance computed the distance between groups and classified the genotype into the group with the smallest squared distance [14]. The distance between the MT group and the S group was much less than that between the T group and the S group.

### 3.3. Genotypic Analysis Based on SSR Markers

#### 3.3.1. Hierarchical Clustering of Genotypes Based on SSR Markers

**Figure 5:**
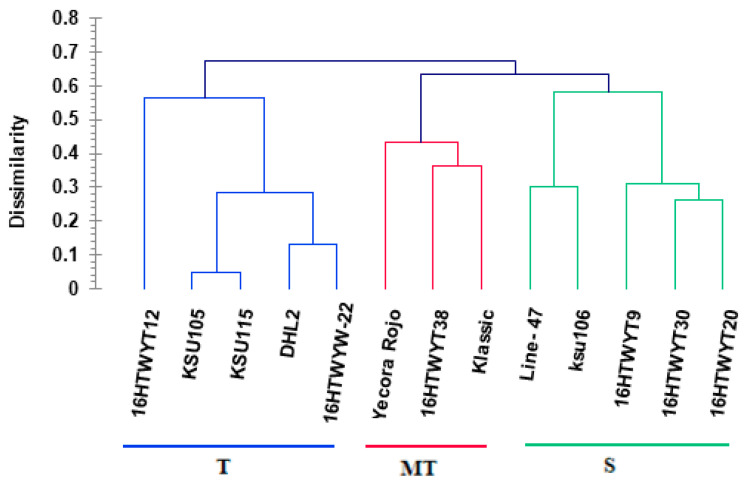
Dendrogram showing the clustering of 13 wheat genotypes based on SSR markers.

HC analysis divided 13 genotypes into three main categories based on allelic data of 27 SSRs linked to drought-tolerant genes (Figure 5). The first category (T, tolerant) covered five genotypes (DHL2, KSU105, 16HTWYT12, 16HTWYW-22, and KSU115). The second category (MT, moderate tolerant) covered three genotypes (16HTWYT-38, Yecora Rojo, and Klassic). The three categories (S, sensitive) covered five genotypes (16HTWYT-30, 16HTWYT-20, 16HTWYT-9, KSU 106, and Line47). The clustering of thirteen genotypes derived from phenotypic distance was associated with genetic distance through the Mantel test, which showed a significant positive correlation (r = 0.271, *p* < 0.018, and alpha = 0.05) between phenotypic distance and genetic distance. These positive correlations between the distance of phenotypic and genetic were necessary, as SSR markers were used as an efficient tool for accessing tolerant genotypes in the early stages of a breeding program.

#### 3.3.2. Association of SSR Markers with Agro-Physio-Biochemical Traits

SMLR analysis was used to determine SSR markers most closely related to agro-physio-biochemical traits under control and drought conditions and DTI. In general, different markers showed a significant association with nine out of thirty agro-physio-biochemical traits studied. The cumulative R^2^ under control conditions ranged from 0.313 for GY to 0.911 for RT; under drought conditions, it ranged from 0.356 for GFD to 0.967 for GB, and DTI ranged from 0.350 for GFD to 0.965 for GB. The marker Wmc326 was significantly linked with five traits (GY, GB, PPO, Chl, RT, and GFD) under drought conditions (R^2^ ranged from 0.321 for RT to 0.623 for GB). It also significantly linked with three traits (GY, GB, and Chl) for DTI (R^2^ ranged from 0.404 for Chl to 0.456 for GB). The marker Wmc65 was significantly linked with the GB trait under control and drought conditions and DTI, as well as with the PH trait under control and DTI (Table 5).

## 4. Discussion

The abiotic stresses consisting of heat, drought, salinity, mineral toxicity, and waterlogging are major agricultural problems, leading to serious wheat yield losses in affected countries [55,56,57]. Recent studies show that drought negatively affects 42% of the wheat production area [58]. Climatic instability and rising water scarcity are predicted in the future, which may lead to significant conversion of mega-productive environments into environments with short-season drought stress [59,60]. These conditions pose a unique challenge to plant breeders and researchers in relevant fields for breeding climatically tolerant genotypes. However, it is difficult to understand drought traits given that it is a polygenic controlled by a large number of genes [2]. Genotyping and recombinant DNA technology increase the knowledge capacity and create valuable tools to assist the selection of desirable varieties such as abiotic stress tolerance with better mean performance for creating new improved generations (genotypes) of sustainable crops [61]. The advantage of the marker-assisted selection (MAS) technique is that the DNA content of a cell is not impacted by the surrounding environment or the age of the plant [1,22,28,62]. However, phenotyping is impacted by the surrounding environment, so it often requires a large set of phenotypic data and multiple seasons for evaluation. In addition, the heterogeneity of agricultural land negatively impacts field evaluation [63]. In this study, 60 genotypes were screened using an SSR marker, and 13 selected genotypes showed varying results in drought tolerance. 

One of the objectives of plant breeding programs is to produce new varieties that cope well with different environmental stresses. We phenotyped the selected genotypes using 30 different agro-physio-biochemicals and 4 quality traits under drought stress compared to the control treatment. Significant differences were found between both conditions, suggesting that genotypes under drought stress were susceptible to a lack of water. The genotypes showed high genetic variation, with different rankings in all traits, which can be leveraged in a phenotypic selection under drought stress and are very popular in quantitative traits [2,6,17]. The interaction was also highly significant for these traits, indicating the need for greater evaluation of the genotypes under various environments and years to examine the drought-related traits. Phenotypic variation in genotypes had a significant role in dominant GEI for all traits (Table 1). The amount of drought tolerance enhancement is determined using levels in the genetic variation and heritability of the traits. The h2 provides information on the variation amount of genetics relative to phenotypic variation, which can be used for predictive validity and the reliability of phenotypic values [64,65,66]. Al-Ashkar et al. [14] and Burton [67] found that the combination of the H2, GCV, and GA gives credible assessments of the expected GG through phenotypic selection. Traits with h2 (>60.0%) and GG (>20.0%) together indicate that the variation is largely due to genetic factors, making them reliable candidates for a selection process [66]. GY alone is poor in predicting the response, so helping other traits can provide a strong indicator of indirectly selecting improved GY under drought stress, helping plant scholars in making more balanced decisions during genotype selection [14]. 

In this study, nineteen traits were considered: six (SL, FLA, DPPH, ChI, Pro, and GI) and 13 (Pn, Gs, Ci, E, PC, WGC, DGC, NSS, RT, CAT, PPO, SOD, and GB) combined, with high h^2^ (>60.0%) and GG (>20.0%) highly for the former and moderate h^2^ (>30.0%) and GG (>10.0%) for the latter, which indicate additive gene effects and can be used as credible screening criteria for assessing genotypic drought tolerance [66,68]. All measured traits showed GCV < PCV, despite breeders’ preference to obtain GCV > PCV [69]. Many scholars used plant traits in breeding programs to evaluate genotypes of drought tolerance [2,6], especially when the traits are genetically stable traits and easy to measure [14,68,70]. Under drought stress, the means of all studied traits were lower than under normal conditions and were more pronounced in sensitive genotypes, unlike many biochemical traits that increased in values. Similar trends have been reported for wheat genotypes [2,21,71]. The traits that respond to drought stress in breeding programs are particularly desirable to evaluate the tolerance of genotypes when the measurement methods are quick, inexpensive, and easy [2,14,70]. PCA is useful in trait reduction, dealing with the problem of multicollinearity, and identifying important traits that are located in the first two components (loadings ≥ 0.381), as presented in Table 2. Tolerant genotypes (16 HWY-22, KSU105, KSU110, and DHL2) under drought exhibited high correlations with most physiological traits (Figure 3). Combining genetic parameters and PCA results, we found six traits (SL, FLA, DPPH, ChI, Pro, and GI) with high values of heritability and genetic gain and were located in the first two components, making these traits efficient screening criteria [2,70]. 

From previous analyses, we used 15 traits for screening drought-tolerant genotypes while excluding 15 traits with low heritability and/or genetic gain not located in PCA1 and PCA2. After dealing with the problem of multicollinearity through PCA, we used outcomes in SMLR, PC, HC, and LD analyses [6]. Drought tolerance is a complex genetic trait and is significantly affected by environmental factors, so the genotypes’ drought tolerance index should not rely solely on GY [2]. We used further statistical analyses to ensure the accuracy of the results; SMLR and PC analyses are instrumental for understanding the dependent correlation with independent variables [68,72]. SMLR results indicated that FLA, Gs, PH, and RT were relevant to GY (R^2^ was 0.988, *p* < 0.0001), and their contribution rates were 0.870, 0.049, 0.056, and 0.014, respectively (Table 3). Since FLA was substantially correlated with yield, we searched for the traits associated with FLA (R^2^ was 0.975, *p* < 0.0001), which were GB, PPO, Chl, and GFD, with contribution values of 0.827, 0.059, 0.048, and 0.040, respectively. We further conducted PC analysis to separate direct and indirect impacts. FLA alone contributed 0.464 as a direct effect on GY, and when the FLA trait was the dependent variable, the GB trait alone contributed 0.437 as a direct effect. A direct effect shows the correlation between the interpreted trait and its direct effectiveness and suggests its use in the selection process [1,73].

The FLA is a valuable trait representing overall plant performance, encompassing radiation use efficiency, photosynthesis, chlorophyll content, and plant competition [74,75]. Genotypes with a high capacity for FLA survival, photosynthesis, and gas exchange under drought stress are highly desirable. FLA is a robust physiological indicator used in wheat breeding programs for identifying drought-tolerant genotypes. Plants synthesize GB in response to abiotic stress, and it plays an important role in osmoregulation by preserving macromolecule’s activity and membrane integrity against stresses while scavenging ROS [76,77]. Using the results from SMLR and PC analyses, we used nine traits (FLA, Gs, PH, RT, GB, PPO, Chl, GFD, and GY) to create an HC analysis with 13 wheat genotypes used to determine genotype categories for drought -tolerance based on a genetic dissimilarity matrix (Figure 4). HC analysis showed three major categories (T, MT, and S) based on the tolerance range of genotypes under drought. The MT and S categories included five genotypes each, while the T category included three genotypes. HC analysis has been used for ranking the drought-tolerant wheat genotypes by many scholars [6,28,29,67,68] without validating their categories. So, we here verified categories through LD analysis, which showed that prior and posterior categories were completely identical in the thirteen genotypes used (Table 4). But cross-validation showed differences in the compatibility proportion of prior and posterior categories, with compatibility observed in nine genotypes (% correct = 69.23%) and incompatible in four genotypes (16HTWYT-30, 16HTWYT-20, 16HTWYT-12, and 16HTWYT-22). Therefore, LD analysis is regarded as a distinct statistical tool (as a selection criterion for accuracy and credibility) for verifying genotype resources of drought tolerance [62,78,79]. 

Assessing drought-tolerance genotypes through multiple agro-physio-biochemical traits may lack accuracy due to the influence of environmental and be costly and time-consuming. To overcome these limitations, a complementary approach was urgently required to combine accurate evaluation with rapid and cost-effective methods. Molecular markers linked to QTLs in MAS are responsible for key agro-physio-biochemical traits under drought stress as SSR markers and are the best tool to detect genotypes with drought tolerance [17,33,62,79]. In this study, HC analysis based on SSR markers categorized 13 genotypes into three main tolerance categories (Figure 5). The complementary approach between phenotyping analysis (phenotypic distance) and genotyping analysis (genetic distance) revealed a significant positive correlation (r = 0.271 and *p* < 0.018) through the Mantel test. These positive correlations are necessary, highlighting SSR markers as an efficient tool for accessing tolerant genotypes in the early stages of a breeding program. Our results reinforce similar findings from many studies [17,78,79,80,81]. Two genotypes (16HTWYT-12 and KSU105) within the categories showed greater distance in the genotyping analysis compared to the categories based on phenotyping analysis, while the genotype (ksu106) showed even greater distancing. Ten genotypes (16HTWYT30, 16HTWYT20, 16HTWYT9, Line-47, DHL2, 16HTWYW-22, KSU115, 16HTWYT38, Yecora Rojo, and Klassic) out of thirteen were completely identical in both analyses of genotyping and phenotyping.

Because of the importance of markers linked to agro-physio-biochemical traits and their role in drought tolerance, we have identified 12 and 11 markers linked to some studied traits under drought conditions and DTI, respectively (Table 5). These findings show that the nine traits (FLA, Gs, PH, GY, GB, PPO, Chl, RT, and GFD) can be used as reliable indicators in the process of selecting wheat genotypes for drought tolerance. The markers (Wmc326, Wmc65, Wmc154, Wmc18, and Cfd9) were highly correlated with various traits, which validates the phenotypic assessment of genotypes [8,82,83,84,85]. These markers hold great potential for future discoveries in breeding programs focused on selecting and producing wheat genotypes with drought tolerance.

## 5. Conclusions

In this study, we studied genetic and phenotypic analyses to detect wheat genotypes with drought tolerance using multivariate analysis techniques. We found six traits that exhibit a combination of high heritability (>60%) and genetic gain (>20%), so it represents a high degree of interest. PC and SMLR analyses together have identified nine traits as a screening tool that effectively detects variations among genotypes, which were used in HC analysis. This analysis divided the 13 genotypes into three main categories, a categorization that was completely confirmed using LD analysis and in ten genotypes through SSR markers. So, the significant association between several mor-pho-physio-biochemical traits and SSR markers was revealed through the Mantel test. The traits and SSR markers identified in our study could be recommended as suitable screening criteria for detecting drought tolerance. Wheat genotypes (DHL2, 16HTWYT-22, and KSU115) could be used as promising genetic sources for drought-tolerant breeding programs. Still, additional efforts are needed to develop new measurement instruments that are rapid, more accurate, and capable of assessments for these traits across numerous genotypes. This area will be the focus of our future studies.

## Figures and Tables

**Table 1:** Analysis of variance for 30 traits estimated in 13 wheat genotypes under drought stress and control.

Source	DF	Pn	Gs	Ci	E	Chl	PC	GI	WGC	DGC	PH	DH	DM	SL	NS	NSS
Rep	2	0.365	0.0002	25.906	0.277	0.004	0.631	12.747	0.616	0.003	3.128	0.051	13.128	0.474	762.859	0.154
I	1	1365.716 **	0.292 **	166,828.1 **	149.66 **	30.688 *	17.033 *	211.207 ^ns^	139.361 **	5.600 **	4063.705 **	714.051 **	5008.013 **	11.538 **	213,938.782 **	149.53 **
Error ^a^	2	0.382	0.00001	103.586	0.188	0.395	0.905	30.756	1.044	0.003	0.359	0.051	13.128	0.115	158.244	1.077
G	12	7.055 **	0.003 **	2131.897 **	0.96 **	0.964 **	6.609 **	555.003 **	43.507 **	4.933 **	167.513 **	32.218 **	150.427 **	14.421 **	3908.987 **	8.872 **
I * G	12	3.004 **	0.001 **	601.333 **	0.431 **	0.215 **	2.573 **	54.762 **	24.021 **	2.043 **	87.205 **	12.218 **	50.568 **	0.344 **	2395.226 **	2.65 **
Error^b^	48	0.632	0.0003	74.685	0.138	0.055	0.721	12.296	1.077	0.021	10.244	0.051	0.517	0.517	425.44	1.004
Genetic Parameters																
σ^2^G		0.675	0.000	255.094	0.088	0.125	0.673	83.374	3.248	0.482	13.385	3.333	16.643	2.346	252.294	1.037
σ^2^e		0.105	0.000	12.448	0.023	0.009	0.120	2.049	0.180	0.004	1.707	0.009	0.086	0.086	70.907	0.167
σ^2^Ph		1.176	0.001	355.316	0.160	0.161	1.102	92.501	7.251	0.822	27.919	5.370	25.071	2.432	651.498	1.479
h^2^ %		57.420	66.667	71.794	55.104	77.697	61.068	90.133	44.788	58.585	47.941	62.077	66.384	96.457	38.725	70.131
G.C.V. %		9.191	7.347	7.662	6.948	13.036	7.579	10.916	9.083	10.185	4.249	2.593	3.698	15.601	3.269	6.507
Ph.C.V. %		12.130	8.998	9.043	9.360	14.789	9.698	11.498	13.573	13.306	6.137	3.291	4.539	15.885	5.253	7.770
GA		1.283	0.031	27.878	0.454	0.642	1.320	17.858	2.484	1.094	5.218	2.963	6.847	3.099	20.362	1.757
GG %		14.348	12.358	13.374	10.625	23.671	12.200	21.348	12.523	16.059	6.060	4.209	6.206	31.563	4.190	11.226
**Source**	**DF**	**GFD**	**GFR**	**TKW**	**GY**	**RT**	**FLA**	**CAT**	**POD**	**PPO**	**SOD**	**DPPH**	**TPC**	**Pro**	**RWC**	**GB**
Rep	2	0.051	0.304	3.962	0.012	0.279	2.665	0.005	0.94	0.007	0.034	0.096	0.004	0.006	0.279	0.079
I	1	1354.167 **	44.192 *	1117.30 **	79.50 **	13,479.04 **	4185.28 **	216.430 **	230.043 **	2.619 **	14.878 **	34,491.283 **	0.180 ^ns^	5.865 **	13,479.05 **	46.066 *
Error ^a^	2	0.051	0.574	3.882	0.067	62.231	4.203	0.2	0.06	0.009	0.024	0.096	0.168	0.002	62.231	0.735
G	12	57.013 **	2.76 **	38.715 **	1.088 **	73.831 **	194.66 **	6.78 **	5.312 **	0.038 **	0.403 **	460.565 **	0.14 *	0.163 **	73.831 **	3.371 **
I * G	12	25.333 **	1.045 **	13.241 **	0.416 **	34.313 **	54.192 **	3.208 **	4.641 **	0.02 **	0.222 **	190.791 **	0.08 **	0.062 **	34.313 **	1.688 **
Error ^b^	48	0.051	0.531	1.645	0.078	3.248	14.302	0.067	0.139	0.001	0.006	8.877	0.045	0.009	3.248	0.185
Genetic Parameters																
σ^2^G		5.280	0.286	4.246	0.112	6.586	23.411	0.595	0.112	0.003	0.030	44.962	0.010	0.017	6.586	0.281
σ^2^e		0.009	0.089	0.274	0.013	0.541	2.384	0.011	0.023	0.000	0.001	1.480	0.008	0.002	0.541	0.031
σ^2^Ph		9.502	0.460	6.453	0.181	12.305	32.443	1.130	0.885	0.006	0.067	76.761	0.023	0.027	12.305	0.562
h^2^ %		55.566	62.138	65.799	61.765	53.525	72.161	52.684	12.632	47.368	44.913	58.575	42.857	61.963	53.525	49.926
G.C.V. %		5.638	3.259	4.890	4.983	14.227	12.019	35.450	10.005	19.542	32.210	14.267	7.109	37.975	3.131	17.304
Ph.C.V. %		7.564	4.135	6.028	6.340	19.446	14.149	48.840	28.150	28.394	48.062	18.641	10.860	48.243	4.280	24.490
GA		3.528	0.868	3.443	0.542	3.868	8.467	1.154	0.245	0.078	0.240	10.572	0.135	0.210	3.868	0.771
GG %		8.658	5.293	8.171	8.067	21.441	21.033	53.006	7.325	27.707	44.467	22.493	9.588	61.580	4.719	25.188

* = significant at *p* ≤ 0.05, ** = significant at *p* ≤ 0.01, ns = insignificant, a = first error and b = second error.

**Table 2:** PCA of 13 wheat genotypes: eigenvalues, proportion, and cumulative variance for the first four components of measured traits under drought.

	PCA1	PCA2	PCA3	PCA4
Eigenvalue	18.769	5.648	1.504	1.016
Variability (%)	62.564	18.827	5.013	3.386
Cumulative %	62.564	81.391	86.404	89.790
Eigenvectors:				
RWC	**0.974**	0.004	0.005	0.0002
RT	**0.974**	0.004	0.0005	0.0002
Chl	**0.931**	0.020	0.003	0.0001
CAT	**0.496**	0.372	0.057	0.016
POD	**0.549**	0.299	0.069	0.000
PPO	**0.485**	0.383	0.085	0.001
SOD	**0.647**	0.275	0.034	0.005
Pro	**0.534**	0.301	0.013	0.0003
TPC	0.069	0.026	0.293	**0.381**
DPPH	**0.646**	0.273	0.036	0.008
GB	**0.417**	0.334	0.0001	0.002
Pn	**0.97**	0.003	0.003	0.001
Gs	**0.959**	0.010	0.012	0.003
Ci	**0.958**	0.021	0.002	0.000
E	**0.978**	0.007	0.000	0.001
FAL	**0.776**	0.100	0.0003	0.001
DH	**0.776**	0.146	0.008	0.005
DM	**0.846**	0.121	0.010	0.003
GFD	**0.822**	0.095	0.011	0.002
GFR	**0.641**	0.040	0.002	0.019
PH	**0.645**	0.001	0.029	0.033
NS	**0.859**	0.074	0.001	0.010
SL	0.187	**0.440**	0.058	0.108
NSS	**0.717**	0.133	0.040	0.014
TWK	**0.817**	0.108	0.003	0.006
GY	**0.876**	0.079	0.005	0.007
WGC	0.098	**0.464**	0.262	0.135
DGC	0.029	**0.515**	0.285	0.131
GI	0.001	**0.589**	0.001	0.109
PC	0.091	**0.410**	0.173	0.173

Abbreviations are as described in Section 2.

**Table 3:** Stepwise regression analyses for grain yield and flag leaf area (dependent index) with seven yield-related traits (independent indices).

	Stepwise Regression					Path Coefficient			
Dependent Variable	Source					Partitioning the Correlation			R^2^
		Regression Coefficient	*p*-Value	R^2^ Par.	R^2^ Com.	Direct Effect	Indirect Effect	Correlation Value	Direct Effect
**GY**	Intercept	18.876							
	FLA	14.247	<0.0001	0.870	0.870	0.681	0.230	0.911	0.464
	Gs	0.790	<0.0001	0.049	0.919	0.340	−0.371	−0.031	0.115
	PH	21.076	0.001	0.056	0.975	0.294	−0.282	0.011	0.086
	RT	−1.151	0.017	0.014	0.988	−0.128	0.278	0.150	0.016
	Total direct effect								0.681
	Total indirect effect								0.307
	Total R^2^				0.988				0.988
	Residual				0.109				0.109
**FAL**	Intercept	0.309							
	GB	−0.319	<0.0001	0.827	0.827	−0.661	−0.240	−0.901	0.437
	PPO	−0.171	0.002	0.059	0.886	−0.257	0.070	−0.186	0.066
	Chl	−0.380	0.005	0.048	0.935	−0.214	0.114	−0.099	0.046
	GFD	−0.150	0.007	0.040	0.975	−0.292	0.792	0.500	0.085
	Total direct effect								0.634
	Total indirect effect								0.341
	Total R^2^				0.975				0.975
	Residual				0.158				0.158

**Table 4:** Posterior probability of membership in drought groupings through linear discriminant analysis.

Genotypes	Classification					Cross-Validation			
	Prior	Posterior	Membership Probabilities			Posterior	Membership Probabilities		
			Pr (MT)	Pr (S)	Pr (T)		MT	S	T
16HTWYT30	S	S	0.000	1.000	0.000	**MT**	1.000	0.000	0.000
DHL2	T	T	0.000	0.000	1.000	T	0.000	0.000	1.000
16HTWYT20	S	S	0.000	1.000	0.000	**M** **T**	1.000	0.000	0.000
16HTWYT38	MT	MT	1.000	0.000	0.000	MT	1.000	0.000	0.000
16HTWYT9	S	S	0.000	1.000	0.000	S	0.000	1.000	0.000
KSU105	MT	MT	1.000	0.000	0.000	MT	1.000	0.000	0.000
16HTWYT12	S	S	0.000	1.000	0.000	**M** **T**	1.000	0.000	0.000
Yecora Rojo	MT	MT	1.000	0.000	0.000	MT	1.000	0.000	0.000
16HTWYT22	T	T	0.000	0.000	1.000	**S**	0.000	1.000	0.000
KSU115	T	T	0.000	0.000	1.000	T	0.000	0.000	1.000
Klassic	MT	MT	1.000	0.000	0.000	MT	1.000	0.000	0.000
Line47	S	S	0.000	1.000	0.000	S	0.000	1.000	0.000
ksu106	MT	MT	1.000	0.000	0.000	MT	1.000	0.000	0.000

Bold letters indicate misclassified wheat genotypes.

**Table 5:** Selection of influential markers (independent variables) with nine studied traits (dependent variable) for control, drought, and tolerant index based on SMLR analysis.

Traits	Treatments	Markres	R^2^ Par.	R^2^ Com.	*p*-Value *
GY	Control	Gwm337	0.336	0.336	0.038
	Drought	Wmc326	0.385	0.385	0.024
	index	wmc326	0.425	0.425	0.016
FLA	Drought	Wmc326	0.460	0.460	0.11
	index	Wmc65	0.359	0.359	0.030
GB	Control	Wmc154	0.579	0.579	0.000
		Cfd1	0.204	0.783	0.012
	Drought	Wmc326	0.623	0.623	0.000
		Wmc503	0.195	0.819	0.001
		Wmc65	0.114	0.933	0.001
		Cfd9	0.034	0.967	0.22
	index	Wmc326	0.456	0.456	0.000
		Wmc65	0.219	0.675	0.000
		Wmc170	0.211	0.886	0.001
		Wmc249	0.049	0.935	0.012
		Wmc405	0.030	0.965	0.043
Gs	Control	Wmc405	0.313	0.313	0.047
PPO	Control	Cfd9	0.389	0.389	0.015
		Gwm369	0.222	0.611	0.038
	Drought	Wmc326	0.458	0.458	0.11
	index	Cfd1	0.434	0.434	0.002
		Gwm369	0.210	0.644	0.036
Chl	Control	Wmc503	0.392	0.392	0.022
	Drought	Wmc326	0.418	0.418	0.017
	index	Wmc326	0.401	0.401	0.005
		Cfd9	0.228	0.629	0.033
RT	Control	Gwm369	0.699	0.699	0.000
		Wmc326	0.159	0.857	0.006
		Wmc154	0.054	0.911	0.044
	Drought	Wmc326	0.321	0.321	0.000
		Cfd18	0.306	0.628	0.000
		Cfd9	0.150	0.778	0.011
		Wmc18	0.108	0.886	0.008
		Wmc154	0.058	0.944	0.031
	index	Gwm369	0.546	0.546	0.017
		Cfd1	0.256	0.801	0.000
		Wmc177	0.121	0.922	0.005
GFD	Drought	Wmc326	0.356	0.356	0.031
	index	Wmc170	0.350	0.350	0.033
PH	Control	Wmc65	0.396	0.396	0.001
		Wmc154	0.334	0.730	0.000
		Wmc74	0.169	0.899	0.002
		Wmc18	0.048	0.920	0.028
	index	Wmc65	0.464	0.464	0.010

Coefficient partial determination (R^2^ Par.), cumulative coefficient determination (R^2^ Com.), * means *p*-value of coefficient partial determination. Abbreviations as described in Section 2.

## Data Availability

All data is contained within the article or Supplementary Materials.

## Supplementary Materials

The following supporting information can be downloaded at https://www.mdpi.com/article/10.3390/life14020183/s1, Table S1: Names, pedigree and Source of the 60 bread wheat genotypes.
