# Supplementary material for: Combining Genetic and Phenotypic Analyses for Detecting Bread Wheat Genotypes of Drought Tolerance through Multivariate Analysis Techniques
Source: Life (Basel). 2024 Jan 25;14(2):183. doi: 10.3390/life14020183 (PMC10890630; doi:10.3390/life14020183)
Supplement: Supplementary file 1 [file life-14-00183-s001.zip › life-2799625-supplementary.pdf]

(Table S1) Names , pedigree and Source of the 60 bread wheat genotypes

Name	Pedigree	Source
16HTWYT-6	BOKOTA/3/KINGBIRD #1//INQALAB 91*2/TUKURU	CIMMYT
16HTWYT-30	SAUAL/YANAC//SAUAL/5/UP2338*2/SHAMA/3/MILAN/KAUZ//CHIL/CHUM18	CIMMYT
16HTWYW-15	AMUR*2/3/TRCH/SRTU//KACHU	CIMMYT
16HTWYW-14	SAUAL/MUTUS/3/KINGBIRD #1//INQALAB 91*2/TUKURU/4/BAJ #1/TECUE #1	CIMMYT
16HTWYW-46	NADI/COPIO//NADI	CIMMYT
16HTWYW-28	SAUAL/YANAC//SAUAL/5/UP2338*2/SHAMA/3/MILAN/KAUZ//CHIL/CHUM18	CIMMYT
16HTWYW-42	FRNCLN/ROLF07/3/KACHU #1/KIRITATI//KACHU/4/FRANCOLIN #1/YANAC	CIMMYT
16HTWYW-50	SUP152/5/CHRZ//BOW/CROW/3/WBLL1/4/CROC_1/AE.SQUARROSA (213)//PGO	CIMMYT
16HTWYW-10	FRET2*2/KUKUNA//PRINIA/PASTOR/3/2*COPIO	CIMMYT
16HTWYW-34	KFA/5/REH/HARE//2*BCN/3/CROC_1/AE.SQUARROSA	CIMMYT
16HTWYW-24	BECARD/AKURI*2/3/PBW343*2/KUKUNA*2//FRTL/PIFED	CIMMYT
16HTWYW-8	SEHER 06/3/PBW343*2/KUKUNA//TECUE #1	CIMMYT
16HTWYW-25	SAUAL*2/6/CNDO/R143//ENTE/MEXI_2/3/AEGILOPS SQUARROSA	CIMMYT
16HTWYW-21	TRCH/SRTU//KACHU*2/5/UP2338*2/SHAMA/3/MILAN/KAUZ//CHIL/CHUM18	CIMMYT
16HTWYW-31	SAUAL/YANAC//SAUAL/3/SUP152/MUU/4/SAUAL/YANAC//SAUAL	CIMMYT
16HTWYW-20	TRCH/SRTU//KACHU*2/5/UP2338*2/SHAMA/3/MILAN/KAUZ//CHIL/CHUM18/4/UP2338*2/SHAMA	CIMMYT
16HTWYW-38	SAUAL/MUTUS/3/ATTILA*2/PBW65*2//KACHU/4/SUP152/AKURI//SUP152	CIMMYT
16HTWYW-9	WHEAR//2*PRL/2*PASTOR/5/UP2338*2/SHAMA/3/MILAN/KAUZ//CHIL/CHUM18	CIMMYT
16HTWYW-5	KACHU #1/YUNMAI 47//KACHU/5/SAUAL/3/C80.1/3	CIMMYT
SAWYT31	WORRAKATTA/2*PASTOR	CIMMYT
16HTWYW-43	FRNCLN/ROLF07//COPIO/3/FRNCLN*2/TECUE #1	CIMMYT
16HTWYW-26	COPIO/7/SAUAL*2/6/CNDO/R143//ENTE/MEXI_	CIMMYT
16HTWYW-32	WBLL1*2/BRAMBLING/4/BABAX/LR42//BABAX*2/3/SHAMA*2/5/PBW343*2/KUKUNA*2//FRTL/PIFED	CIMMYT
16HTWYW-41	WAXWING/KIRITATI*2/3/C80.1/3*BATAVIA//2	CIMMYT

16HTWYW-23	SAUAL/4/CROC_1/AE.SQUARROSA (205)//KAUZ/3/ATTILA/5/SAUAL/8/TACUP	CIMMYT
16HTWYW-32	QUAIU #1/SUP152	CIMMYT
16HTWYW-27	COPIO/7/SAUAL*2/6/CNDO/R143//ENTE/MEXI_	CIMMYT
16HTWYW-11	WBLL1*2/4/YACO/PBW65/3/KAUZ*2/TRAP//KAUZ/5/SAUAL/6/2*KINGBIRD #1//INQALAB 91*2/TUKURU	CIMMYT
16HTWYW-29	SAUAL/YANAC//SAUAL/5/UP2338*2/SHAMA/3/MILAN/KAUZ//CHIL/CHUM18/4/UP2338*2/SHAMA/6/UP2338 *2/SHAMA/3/MILAN/KAUZ//CHIL/CHUM18/4/UP2338*2/SHAMA	CIMMYT
SAWYT42	AMUR/3/KINGBIRD #1//INQALAB 91*2/TUKURU/4/AMUR	CIMMYT
16HTWYW-12	CMSS11Y00824T-099TOPM-099Y-099M-099NJ-099NJ-28WGY-0B	CIMMYT
16HTWYW-39	AMUR/3/KINGBIRD #1//INQALAB 91*2/TUKURU/4/AMUR	CIMMYT
16HTWYW-2	CMSS06B00734T-099TOPY-099ZTM-099Y-099M-13WGY-0B	CIMMYT
16HTWYW-22	CMSS11Y00977T-099TOPM-099Y-099M-099NJ-099NJ-16WGY-0B	CIMMYT
Yecora Rojo	The wheat line Yecora Rojo, developed by the International Wheat and Maize Improvement Centre (CIMMYT, Mexico)	(CIMMYT
Line 277	Yecora Rojo *KSU110-277	Prof. Abdullah Al-Doss
Line 4	Yecora Rojo *Lang-4	Prof. Abdullah Al-Doss
Line 26	DH-H3-26/ klassic/ku105	Prof. Abdullah Al-Doss
KSU105	HD21-72/RI-474	Prof. Abdullah Al-Doss
Line 25	H4-25 Yecora Rojo *KSU106	Prof. Abdullah Al-Doss
Line190	Yecora Rojo *KSU110-190	Prof. Abdullah Al-Doss
Line 87	Lang*ksu105-87	Prof. Abdullah Al-Doss
KSU 114	Sama / Yecora Rojo L11-23	Prof. Abdullah Al-Doss
Line 76	DH-H3-76 Klassic/KSU105	Prof. Abdullah Al-Doss
KSU110	Sama/ Yecora Rojo L11-6	Prof. Abdullah Al-Doss
Line 240	Yecora Rojo /KSU110-240	Prof. Abdullah Al-Doss
KSU115	Sama/ Yecora Rojo L11-21	Prof. Abdullah Al-Doss
Line 15	Yecora Rojo *lang-15	Prof. Abdullah Al-Doss
Line 30	Yecora Rojo *lang-30	Prof. Abdullah Al-Doss
Line 213	klassic*ku105-213	Prof. Abdullah Al-Doss
Klassic	American Commercial Cultivator	Prof. Abdullah Al-Doss

Lang	Australian Commercial Cultivator	Prof. Abdullah Al-Doss
Line 11	klassic*ku105-11	Prof. Abdullah Al-Doss
Line47	Lang*ksu105-47	Prof. Abdullah Al-Doss
Line 60	Yecora Rojo *lang-60	Prof. Abdullah Al-Doss
Line 66	Yecora Rojo *lang-66	Prof. Abdullah Al-Doss
ksu106	Barouk /R1474-75-3-53-3-3,	Prof. Abdullah Al-Doss
SIDS 1	HD2172/PAVON"S"//1158.57/MAYA74"S"	Agricultural Research Center -Egypt
Sakh94	Opata/Rayon//SITE/MO/3/CHIN/AEGILOPS SQUARROSA(SAUT)//BCN/4/WBLL1	Agricultural Research Center -Egypt
DHL2	Derived from the cross (Giza-164× Giza-168)	Prof. Ibrahim Al-Ashkar
